# Pulse Dye Laser Therapy Successful for Elastosis Perforans Serpiginosa

**DOI:** 10.1155/2019/2860746

**Published:** 2019-12-24

**Authors:** Giulia Rinaldi, Alisha Chacko, Samira Batul Syed

**Affiliations:** ^1^Guys and St. Thomas' Hospital, NHS Foundation Trust, London, UK; ^2^Great Ormond Street Hospital, NHS Foundation Trust, London, UK

## Abstract

**Background:**

We describe a case of elastosis perforans serpiginosa and its successful management with PDL laser.

**Case Presentation:**

A 15-year-old male presented with a history of itchy, raised, red and unsightly lesions on the back of his neck. He was diagnosed with Elastosis Perforans Serpiginosa on tissue biopsy and underwent pulse dye laser therapy over four years with excellent results.

**Conclusions:**

Our results show that pulse dye laser therapy is a safe and effective treatment for elastosis perforans serpiginosa.

## 1. Background

Elastosis perforans serpingosa (EPS) is one of four classic perforating disorders including Kyrle's disease, reactive perforating collagenosis and perforating folliculitis [1]. Histological evaluation allows perforating disorders to be classified according to which skin material appears to be eliminated. In EPS, this involves trans epithelial elimination which is characterised by abnormal dermal elastic, other connective tissue, and cellular elements being extruded from the papillary dermis through the epidermis [2]. The pathogenesis of EPS is divided into three subtypes; idiopathic (where no cause can be identified), reactive (associated with connective tissue disorders such as Marfan's, Ehler Danlos, Down's syndrome or osteogenesis imperfecta) and drug-induced (penicillamine being the commonest drug reported) [3, 4]. EPS usually presents as small umbilicated or keratotic erythematous papules which cluster together in a serpiginous pattern around the neck, face or arms. The appearance of EPS is commonest in males under the age of 30.

## 2. Case Presentation

We present a 15 year old male who was referred with idiopathic EPS presenting as extensive umbilicated papules on his posterior neck present for over 2 years. The patient's main concern was their appearance causing him embarrassment at school.

**Figure 1:**
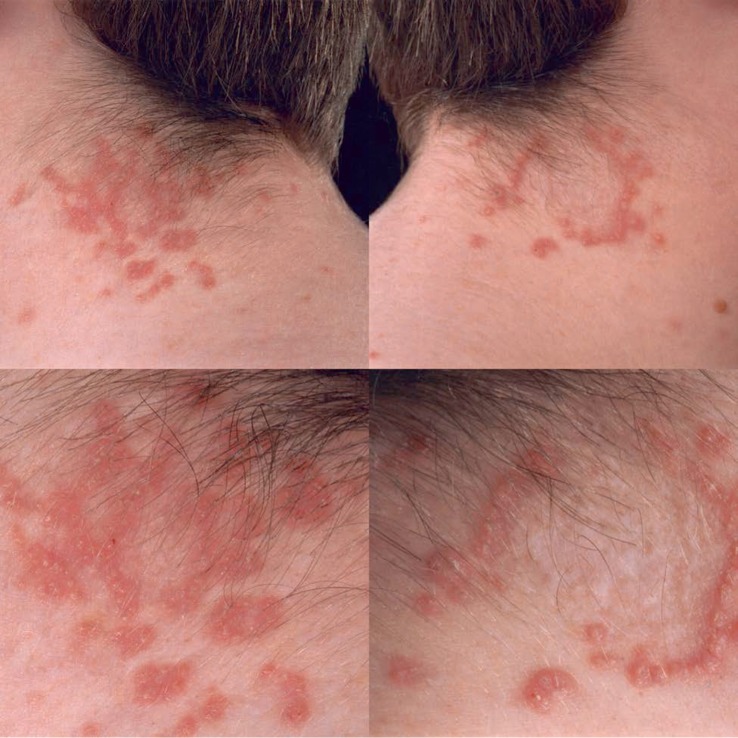
Lesions on left & right neck before treatment.

**Figure 2:**
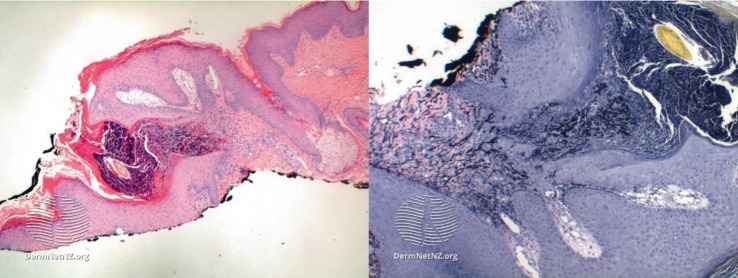
EPS biopsy—https://creativecommons.org/licenses/by-nc-nd/3.0/nz/legalcode.

On examination, he had two localized areas of erythematous umbilicated papules arranged in a serpiginous pattern on both the left and right side of his posterior neck (Figure 1). A biopsy had been performed at his previous centre of care and a diagnosis of EPS was confirmed. An EPS biopsy, as shown on DermNet.nz, shows keratotic debris columns perforating through a hyperplastic epidermis. This can be accentuated using an Elastic-Van Gieson stain that highlights elastic fibres in black (Figure 2) [5].

The patient's main concerns were extreme itch and the unsightly appearance of the lesions. At the time of referral, he had already failed treatment with cryotherapy, topical retinoids and 10% urea cream. The decision was made to treat with pulse dye laser (PDL) therapy.

**Figure 3:**
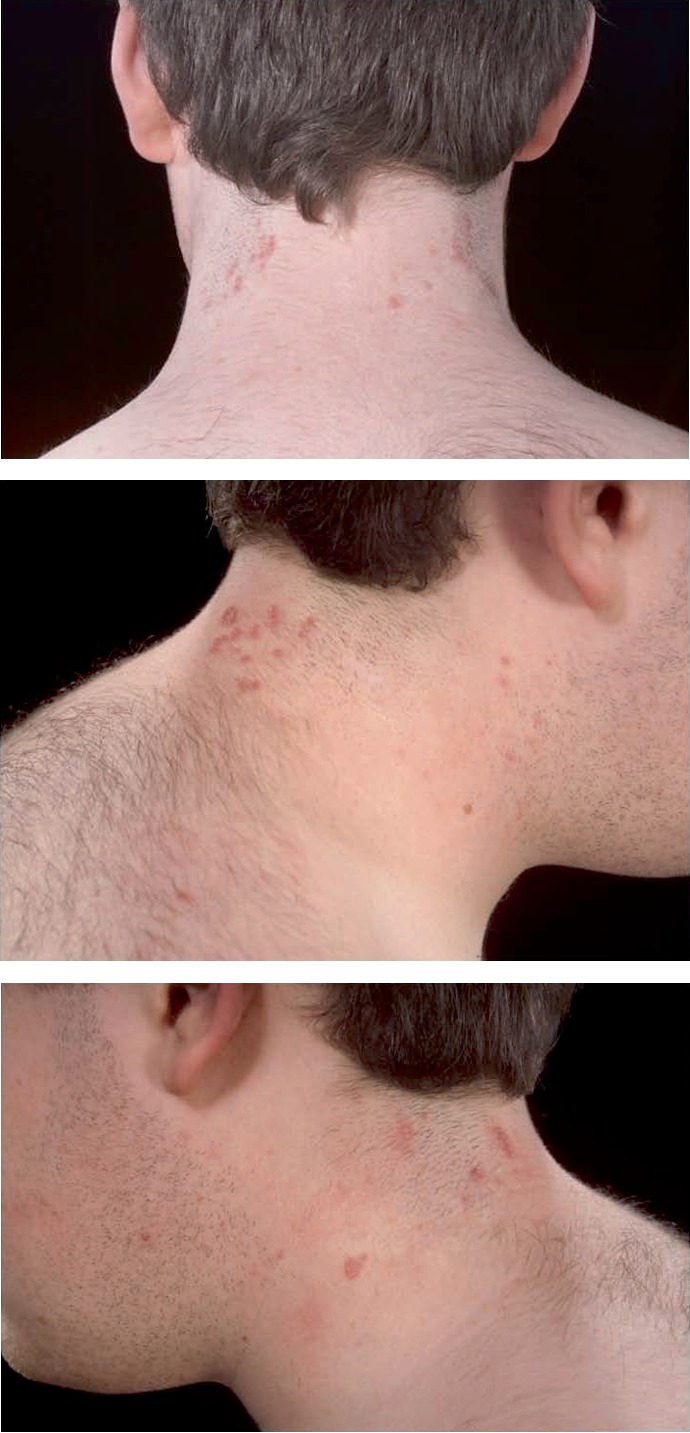
Lesions on left and right neck after 7 laser sessions.

He received eight treatment sessions over a four year period using pulse dye laser of 585 nm wavelength, the parameters of which are shown in Table 1. He achieved positive results with over 75% clearance of the lesions, a significant reduction in itch and colour and flattening of most of the remaining lesions (Figure 3). No side effects were noted by the patient, parents or laser team. Fifteen years after being discharged the patient has received no further treatments for this condition and he has remained free of new lesions.

## 3. Discussion

EPS remains one of the rarer dermatological perforating disorders for which the exact prevalence remains unestablished. Published cases of EPS mainly focus on the association with possible triggers, for example, Down's syndrome, penicillamine, scabies, osteogenesis imperfecta, and systemic sclerosis [3, 4, 6–8]. For less experienced clinicians it can create a diagnostic conundrum and therefore remains best diagnosed via a biopsy and histological examination.

Importantly, EPS remains difficult to treat with minimal published evidence and no consensus on successful treatment methods. Various attempted treatment methods have been reported with many clinicians initially trialling topical steroids and topical retinoids. However these treatments have not shown to be successful [7]. Other treatment options include oral isotretinoin, intralesional steroids, curettage & cautery, and cryotherapy. Similarly these interventions have not been shown to be successful [2, 3, 7, 9]. Experimental treatments such as narrow band UVB, topical imiquimod and tazarotene have shown contradictory results with temporary improvement in certain patients [2, 10]. However in other patients either no improvement was reported or there was rebound flaring upon discontinuation [11, 12].

Over the last three decades laser therapy has emerged as a possible treatment option. One case, reported by Kaufman in the year 2000, demonstrated marked improvement of EPS after PDL therapy (585 nm and fluencies of 6.0–7.0 J/cm^2^) [13]. However, we are not aware of any further cases reported. More recently, the use of CO_2_ laser has had contradictory results with one case reporting it as successful [11] and two other cases reporting clinically insignificant results and even scarring in one patient [12, 14].

As far as we are aware only one other case, almost 20 years ago, has been reported to use PDL laser therapy for EPS treatment. Our experience confirms Kaufman's initial success with PDL laser for treatment of EPS and encourages other practitioners who encounter EPS to consider this treatment method. Furthermore, we encourage fellow laser practitioners to report their experiences in treating this condition. This would produce a larger evidence base and facilitate the creation of a consensus for the successful treatment of this recalcitrant skin condition.

## 4. Conclusions

In conclusion, we report a case of idiopathic EPS that was successfully managed with PDL therapy. This is important as it reconfirms that PDL therapy is a safe and effective treatment for EPS.

## Figures and Tables

**Table 1:** Laser parameters used to treat our patient.

Probe	7 mm
Laser fluence	8–15 J/cm^2^
Pulse duration	0.45–1.5 ms
Maximum pulses	46
Cooling method	Cryogen spray
